# The Role of Small Extracellular Vesicles in Viral-Protozoan Symbiosis: Lessons From *Trichomonasvirus* in an Isogenic Host Parasite Model

**DOI:** 10.3389/fcimb.2020.591172

**Published:** 2020-11-05

**Authors:** Yashini Govender, Tiffany Chan, Hidemi S. Yamamoto, Bogdan Budnik, Raina N. Fichorova

**Affiliations:** ^1^Laboratory of Genital Tract Biology, Department of Obstetrics, Gynecology and Reproductive Biology, Harvard Medical School, Brigham & Women’s Hospital, Boston, MA, United States; ^2^Mass Spectrometry and Proteomics Resource Laboratory, FAS Division of Science, Harvard University, Cambridge, MA, United States

**Keywords:** *Trichomonasvirus*, *T. vaginalis*, exosomes, extracellular vesicles, immune modulation, cytokines, proteomics

## Abstract

The protozoan parasite *Trichomonas vaginalis* (TV), exclusively adapted to the human genital tract, is one of the most common sexually transmitted pathogens. Adding to the complexity of the host-pathogen interactions, the parasite harbors TV-specific endosymbiont viruses (*Trichomonasvirus*, TVV). It was reported that small extracellular vesicles (sEVs) released by TV play a role in host immunity; however, the role of the viral endosymbiosis in this process remained unknown. We hypothesized that the virus may offer evolutionary benefit to its protozoan host at least in part by altering the immunomodulatory properties of sEVs spreading from the site of infection to non-infected immune effector cells. We infected human vaginal epithelial cells, the natural host of the parasite, with TV natively harboring TVV and an isogenic derivative of the parasite cured from the viral infection. sEVs were isolated from vaginal cell culture 24 h post TV infection and from medium where the isogenic TV strains were cultured in the absence of the human host. sEVs from TVV-negative but not TVV-positive parasites cultured alone caused NF-κB activation and increase of IL-8 and RANTES expression by uterine endocervical cells, which provide innate immune defense at the gate to the upper reproductive tract. Similarly, mononuclear leukocytes increased their IL-8, IL-6 and TNF-α output in response to sEVs from virus-negative, but not isogenic virus-positive parasites, the latter exosomes being immunosuppressive in comparison to TV medium control. The same phenomenon of suppressed immunity induced by the TVV-positive compared to TVV-negative phenotype was seen when stimulating the leukocytes with sEVs originating from infected vaginal cultures. In addition, the sEVs from the TVV-positive infection phenotype suppressed immune signaling of a toll-like receptor ligand derived from mycoplasma, another frequent TV symbiont. Quantitative comparative proteome analysis of the secreted sEVs from virus-positive versus virus-negative TV revealed differential expression of two functionally uncharacterized proteins and five proteins involved in Zn binding, protein binding, electron transfer, transferase and catalytic activities. These data support the concept that symbiosis with viruses may provide benefit to the protozoan parasite by exploiting sEVs as a vehicle for inter-cellular communications and modifying their protein cargo to suppress host immune activation.

## Introduction

The extracellular protozoan parasite *Trichomonas vaginalis* (TV) causes trichomoniasis, one of the most common sexually transmitted infections affecting over 200 million men and women each year (Patel et al., 2018). The infection is recurrent, asymptomatic in more than half of those diagnosed and often lasting over a long period of time in the urogenital tract while causing a myriad of complications including infertility, preterm birth, low birth weight, bacterial vaginosis (BV), and increased risk of cancer, human papillomavirus (HPV) persistence, and HIV infection (Fichorova, 2009; Kissinger, 2015; Meites et al., 2015). The poor understanding of the molecular mechanisms used by this parasite to avoid clearance by a robust inflammatory immune response is a critical barrier to prevention of TV-associated health risks and conditions. Adding to the immunological complexity, the majority of the TV clinical isolates stably harbor endosymbiont double-stranded (ds) RNA viruses from the genus *Trichomonasvirus* (TVV) (Goodman et al., 2011a; Goodman et al., 2011b; Fichorova et al., 2017). TVV shed by the parasite is sensed by the toll-like receptor (TLR)-3 in female genital tract epithelial cells where it can induce selective virus-stress response (Fichorova et al., 2012), which however appears insufficient to cause clinically effective inflammatory response to clear the parasite (Graves et al., 2019). Consequently, other molecular mechanisms may be built into the virus-protozoan symbiotic relationship to counteract immune activation and to provide evolutionary advantage to the parasites harboring the virus. We hypothesized that the virus may offer an evolutionary benefit to its protozoan host by altering the immunomodulatory properties of small extracellular vesicles (sEVs) spreading from the site of infection. sEVs are nano-sized membrane-bound particles (<150 nm) which contain a mix of exosomal and non-exosomal vesicles (Mastoridis et al., 2018). sEVs are universally released into mucosal secretions and the blood circulation by both host cells and eukaryotic parasites and mediate cell-cell communications through the sEV molecular cargo and signal transduction (Coakley et al., 2015). However, the role of sEVs in subversion of host immunity by viral-protozoan symbiosis in trichomoniasis has not been elucidated to date.

## Methods

### *T. vaginalis* Culture

The TVV positive TV clinical isolate 347V+ and its TVV-cured derivative 347V- were provided by John F. Alderete (University of Texas Health Science Center, San Antonio, TX) (Wang et al., 1987). The progeny strain 347V- was rendered virus-negative after continuous passage (Benchimol et al., 2002). For some experiments, we also used the TVV negative laboratory strain B7RC2 (ATCC^®^ 50167™) (Fichorova et al., 2012), and the vaginal isolates were previously characterized for their naturally occurring TVV status including UR1 (TVV positive), isolated from the University of Rochester STI Clinic (Goodman et al., 2011a), OC7 (TVV negative) and OC8 (TVV positive), isolated at the Onondaga County Health Department STI Clinic (Syracuse, NY) (Fichorova et al., 2012). Parasites were cultured in modified Diamond’s trypticase-yeast extract-maltose medium supplemented with 10% horse serum (TV medium) at a concentration of 4 × 10^5^ TV/ml at 35°C under anaerobic conditions as described (Fichorova et al., 2012). We did not deplete sEVs from the TV medium prior to culture because we observed suboptimal growth of the parasites in sEV-depleted medium (**Supplementary Figure S1**). To account for sEVs contributed by the horse serum and other medium supplements, we isolated and tested in each experiment sEVs from the complete culture medium alone, which served as a control to culture supernatants after allowing TV to grow for 24 h.

### Vaginal Epithelial Cell Culture and *T. vaginalis* Infection

Human vaginal epithelial cells (Vk2/E6E7) were cultured in modified antibiotic-free, keratinocyte serum-free medium (KSFM) as described (Fichorova et al., 1997). For infection experiments, TV was harvested in late log phase (24 h) by centrifugation (1,000 × g for 5 min) and the TV pellet was re-suspended in KSFM at 4 × 10^5^ TV/ml. Vk cells grown to 80–90% confluency were infected and incubated under anaerobic conditions on a shaker at 50 rpm and 35°C for 24 h, as described (Fichorova et al., 2012).

### Isolation of Small Extracellular Vesicles

Supernatants from 24 h (i) 10 × 10^6^ TV monoculture (ii) Vk + 10 × 10^6^ TV co-culture and (iii) medium alone (TV medium or KSFM) were centrifuged at 2,000 × g for 30 min and passed through a 0.22 µm filter. sEVs were isolated using the Total Exosome Isolation Reagent for cell culture medium (Invitrogen, Carlsbad, CA) per manufacturer’s protocol. The sEV pellets were re-suspended in either PBS or KSFM medium. Exosome Spin Columns (Invitrogen, Carlsbad, CA) were used to remove low molecular weight contaminants (MW<3000) from sEV samples per manufacturer’s protocol. Once purified, samples were sterilized through 0.22 µm filters and frozen at -20°C until use. The sEV size was confirmed by nanotracking. Briefly, sEV preparations were diluted 1000× and 50,000× in PBS and quantified using the ZetaView^®^ (Particle Metrix, Meerbusch, Germany) by translational diffusion size distribution. Nanotracking analysis confirmed presence of sEVs from TV cultures in the peak size range of 76.6–106.9 nm similar to those previously reported for *T. vaginalis* (Twu et al., 2013; Olmos-Ortiz et al., 2017; Nievas et al., 2018) and at peak concentrations ranging from 1.1E9–1.65E11 particles/ml (**Supplementary Figure S2A**). Transmission electron microscopy with immunogold labelling of CD63 confirmed the presence of exosomes in the sEV samples (**Supplementary Figure S2B**).

### sEV Treatment of Non-Infected Bystander Cells

Human endocervical epithelial cell (End1/NFκB) were cultured in KSFM. Cells were seeded into 96-well flat-bottom plates at a density of 5 × 10^5^ cells/ml. sEV treatment dose was calculated based on the sEV equivalent of the parasite load applied to the vaginal infection model. Blood was obtained from five healthy anonymous donors, after written informed consent, at Research Blood Components LLC (Brighton, MA). Peripheral blood mononuclear cells (PBMCs) were isolated using Ficoll^®^-Paque PLUS (GE Healthcare, Uppsala, Sweden) density gradient separation as per manufacturer’s protocol. PBMCs were cultured in RPMI-1640 medium with L-glutamine (Corning™, Manassas, VA) and 10% heat inactivated newborn calf serum (Gibco™, Life Technologies, Grand Island, NY) at a density of 2 × 10^6^ cells/ml in round-bottom 96-well plates. PBMCs were treated with sEVs for 24 h in the presence or absence of 25 nM TLR2/TLR6 ligand mycoplasma-derived macrophage-activating lipopeptide-2 (MALP-2) (Enzo Life Sciences, NY).

### NF-κB Activity

A stably transfected endocervical epithelial cell line, which expresses NF-κB firefly luciferase reporter (End1/NF-κB) previously generated in our laboratory (Singh et al., 2009) was used for measurement of NF-κB activation as described (Fichorova et al., 2012). After 24 h of exposure to sEVs, cells were lysed with GloLysis buffer (Promega, Madison, WI) and luciferase activity was determined using the Bright-Glo Luciferase Assay System (Promega, Madison, WI) as per manufacturer’s protocol.

### Biomarkers of Immune Modulation

Levels of soluble immune mediators were measured in cell supernatants using electrochemiluminescence multiplex assays on a Sector Imager S600 (Meso Scale Discovery (MSD), Gaithersburg, MD). IL-8 and RANTES were measured simultaneously in epithelial culture supernatants by an MSD 2-plex. IL-8, IL-6, IL-10, and TNF-α were measured simultaneously in PBMC supernatants using an MSD multiplex.

### Cell Viability

Epithelial and PBMC cell viability were assessed by the non-radioactive CellTiter96 MTT (3-(4,5-dimethylthiazol-2-yl)-2,5-diphenyltetrazolium bromide) assay (Promega, Madison, WI) and TV viable counts were obtained microscopically with the trypan blue exclusion test (Fichorova et al., 2012).

### sEV Proteomic Analysis

Secreted sEVs were isolated from TV medium, three strains of virus negative TV (347V-, B7RC2 and OC7) and three strains of virus positive TV (347V+, UR1, and OC8). sEV pellets were transferred into Covaris^®^ microTUBE- 15 (Woburn, MA) microtubes with Covaris^®^ TPP buffer. Samples were lysed in Covaris S220 Focused-ultrasonicator instrument with 125W power over 180s with 10% max peak power. Lysed samples were then chloroform/MeOH precipitated and weighed then digested *via* filter aided sample preparation (FASP) digest. Promega^®^ Sequencing Grade Trypsin was used for an overnight digestion at 38 °C. Tandem-Mass-Tag (TMT) peptide labeling was performed using Thermo Scientific TMT Reagents (Thermo Fisher Scientific, San Jose, CA). The pooled sample was fractionated into 10 fractions and submitted for LC-MS/MS experiment that was performed on a Orbitrap Lumos (Thermo Fisher Scientific, San Jose, CA) equipped with Ultimate 3000 (Thermo Fisher Scientific, San Jose, CA). Peptides were trapped onto a 150 µm inner diameter microcapillary trapping column packed first with approximately 3 cm of C18 Reprosil resin (5 µm, 100 Å, Dr. Maisch GmbH, Germany) followed by 50 cm micro Pillar Array Columns (µPAC™) analytical column (PharmaFluidics, Belgium). Separation was achieved through applying a gradient from 5–27% ACN in 0.1% formic acid over 90 min at 200 nl min^−1^. Electrospray ionization was enabled through applying a voltage of 1.8 kV using a home-made electrode junction at the end of the microcapillary column and sprayed from stainless still needle (PepSep, Denmark). The LTQ Orbitrap Lumos was operated in data-dependent mode for the mass spectrometry methods. The mass spectrometry survey scan was performed in the Orbitrap in the range of 395–1,800 m/z at a resolution of 6 × 10^4^, followed by the selection of the twenty most intense ions (TOP20) for CID-MS2 fragmentation in the Ion trap using a precursor isolation width window 2 Da for CID scans, AGC setting of 10,000, and a maximum ion accumulation of 200 ms. Singly charged ion species were not subjected to fragmentation. Normalized collision energy was set to 35 V and an activation time of 10 ms. Ions in a 10 ppm m/z window around ions selected for MS2 were excluded from further selection for fragmentation for 60 s. The same TOP20 ions were subjected to HCD MS2 event in Orbitrap part of the instrument. The fragment ion isolation width was set to 0.7 m/z, AGC was set to 50,000, the maximum ion time was 200 ms, normalized collision energy was set to 27V and an activation time of 1 ms for each HCD MS2 scan. Raw data were submitted for analysis in Proteome Discoverer 2.4 (Thermo Scientific, CA) software. Assignment of MS/MS spectra was performed using the Sequest HT algorithm by searching the data against a protein sequence database including all entries from Uniprot_Human2018_SPonly, Uniprot_Trichomonas_vaginalis, Uniprot_ Saccharomyces_cerevisiae databases, and other known contaminants such as human keratins and common lab contaminants. For the peptide screening, we included the yeast database because TV medium contains yeast extracts. Sequest HT searches were performed using a 20 ppm precursor ion tolerance and requiring each peptides N-/C termini to adhere with Trypsin protease specificity, while allowing up to two missed cleavages. 11-plex TMT tags on peptide N termini and lysine residues (+229.162932 Da) was set as static modifications, while methionine oxidation (+15.99492 Da) was set as variable modification. A MS2 spectra assignment false discovery rate (FDR) of 1% on both protein and peptide level was achieved by applying the target-decoy database search. Filtering was performed using a Percolator (64bit version, reference 6). For quantification, a 0.02 m/z window centered on the theoretical m/z value of each of the six reporter ions and the intensity of the signal closest to the theoretical m/z value were recorded. Reporter ion intensities were exported in result file of Proteome Discoverer 2.2 search engine as an excel table.

The raw data are available at https://massive.ucsd.edu/ProteoSAFe/dataset.jsp?task=d972dafadff24186af5c36bd6b171f11

### Bioinformatics and Statistical Analyses

NFκB activation, MTT and cytokine data were analyzed by analysis of variance (ANOVA) with Tukey’s multiple comparisons test (GraphPad Prism, v.7) and p values <0.05 were considered significant. For quantitative proteomics, principal component analysis and differential expression analysis was performed using homemade R package. Data sets underwent two-way ANOVA analysis and proteins with p values <0.05 were considered significant for down or upregulated hits. **Supplementary Table S1** contains the protein list of TV phenotype 1 (virus-negative) vs. phenotype 2 (virus-positive) with statistics and the gene ontology (GO) analysis.

## Results

### sEVs Derived From TVV-Negative but Not TVV-Positive Parasites Activated NF-KB and Cytokine Production by Uninfected Bystander Epithelial Cells and Effector Immune Cells

Previous evidence showed that sEVs from TV laboratory strains modulate innate immunity in human cervical cells (Twu et al., 2013). We showed using multiple clinical TV isolates that TVV induced NF-kB activation and cytokine upregulation in endocervical, ectocervical and vaginal human epithelial cells (Fichorova et al., 2012). Therefore, to decipher the effect of TVV on TV sEV-mediated immunity, we first treated the same endocervical cells tested previously with sEVs derived from a native TVV positive TV strain (347V+) and its TVV negative isogenic derivative (347V-), and measured NF-κB activation and levels of IL-8 and RANTES. IL-8 was chosen because it is the most abundantly expressed epithelial chemokine for neutrophils involved in clearing protozoan infection (Mercer et al., 2018). RANTES was chosen because its levels are low at baseline in the epithelial cells but are significantly upregulated in response to dsRNA, including TVV genomic dsRNA upon exposure to both purified RNA and whole intact virions (Fichorova et al., 2012). Thus, the RANTES response served as a control for viral contamination of the sEV preparations from the TVV+ strain.

**Figure 1:**
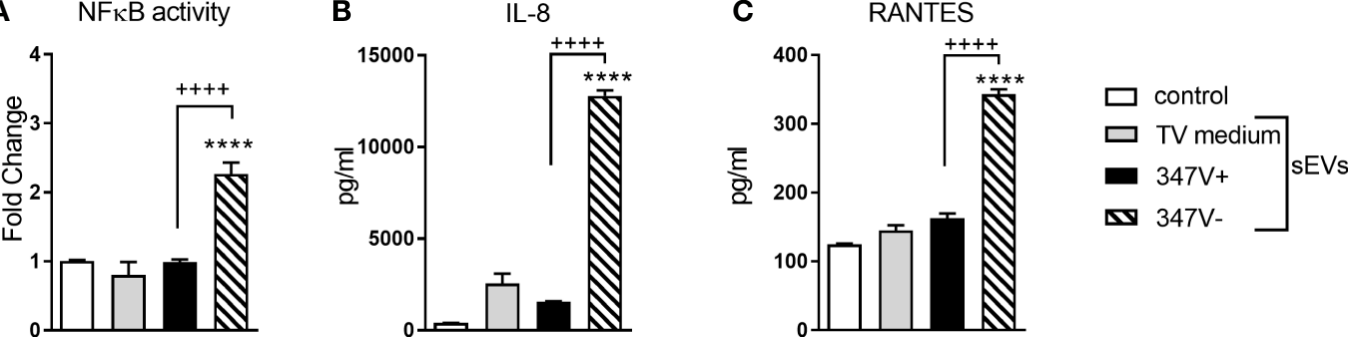
TVV infection status modifies the immunostimulatory properties of TV sEVs in bystander cervical cells. Endocervical cells were treated with control no sEV, sEVs derived from TV medium alone, 347V+, or 347V- for 24 h. **(A)** NFkB activity was measured by a luciferase reporter assay and shown as fold change from control. **(B)** IL-8 and **(C)** RANTES protein levels by MSD assays. Data represent two independent experiments performed in triplicate and plotted as mean ± standard deviation. ****p < 0.0001 represent sEVs different from control. ^++++^p < 0.0001 represent 347V- different from 347V+.

The endocervical cells responded to the sEVs from 347V- but not 347V+ with significant NF-κB activation (>2-fold increase over baseline, p < 0.0001) and increased levels of both IL-8 (>20-fold, p < 0.0001) and RANTES (~2-fold, p < 0.0001) (**Figure 1**). The lack of RANTES response to the 347V+ sEVs confirmed lack of dsRNA contamination of the sEV preparation.

**Figure 2:**
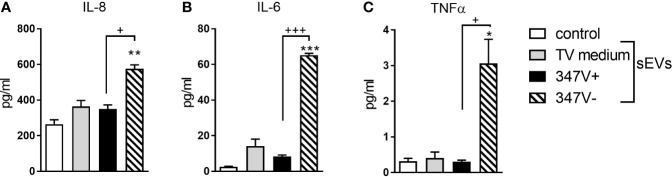
sEVs from virus-negative TV but not virus-positive parasites induce multi-cytokine response in immune cells. PBMCs were treated with control no sEV, sEVs derived from TV medium, 347V+, and 347V- TV strains for 24 h. **(A)** IL-8, **(B)** IL-6, and **(C)** TNF-α protein levels were measured by MSD assays. Graphs represent data from PBMC donor X in duplicate and plotted as mean ± standard error of mean. Results are shown as protein levels normalized to cell viability. *p < 0.05; **p < 0.01; and ***p < 0.001 represent sEVs different from control. ^+^p < 0.05 and ^+++^p < 0.001 represent 347V- different from 347V+.

We next treated PBMCs with 347V+ and 347V- sEVs and measured levels of IL-8, IL-6 and TNFα as markers of leukocyte activation. None of the sEV preparations reduced leukocyte viability below 100% of the medium control (data not shown). 347V- sEVs significantly increased levels of all three cytokines compared to both baseline and 347V+ sEVs, while 347V+ sEVs had no significant effect (**Figure 2** and **Supplementary Figure S3**).

### sEVs Derived From TV Infected Vaginal Cells Activated Leukocytes in a Manner Dependent on the TVV Status of the Parasite

In the natural course of infection, both the human host cells and the extracellular parasite adherent to them can contribute to the sEV pool released by the infected vaginal epithelium. To determine whether the TVV endosymbiont will continue to exert its immunomodulatory effect in the context of the host-parasite sEV pool, we isolated sEVs from the vaginal supernatants in our vaginal TV infection model. In this model in addition to the 347V+ and 347V- isogenic pair, we used a second previously well-characterized TVV+ strain UR1 (Goodman et al., 2011a; Goodman et al., 2011b; Fichorova et al., 2012) and also stimulated the vaginal epithelium with synthetic dsRNA poly(I:C) to mimic the TVV dsRNA effects in the absence of TV.

**Figure 3:**
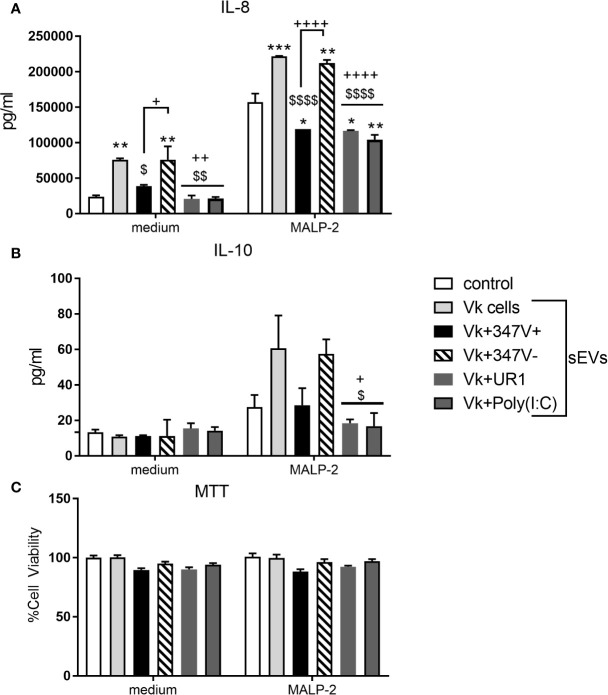
sEVs from TV-infected vaginal cells regulate cellular immunity in a manner dependent on the viral status of the parasite. PBMCs were treated with control no sEV, sEVs derived from vaginal cells (Vk cells), Vk+347V+, Vk+347V-, Vk+UR1, and Vk+Poly(I:C) with or without MALP-2 for 24 h. **(A)** IL-8 and **(B)** IL-10 protein levels were measured by MSD assay. **(C)** MTT assays were performed to confirm cell viability after 24 h treatment relative to control. Graphs represent data from PBMC donor A in duplicate and plotted as mean ± standard error of mean. *p < 0.05; **p < 0.01; and ***p < 0.001 represent sEVs different from control. ^$^p < 0.05; ^$$^p < 0.01; and ^$$$$^p < 0.0001 represent comparisons different to Vk cells sEVs. +p < 0.05; ++p < 0.01; and ^++++^p < 0.0001 represent comparisons different to Vk+347V- sEVs.

As shown in **Figure 3A**, sEVs from the vaginal infection with both TVV+ strains suppressed IL-8 production by the PBMCs in comparison to sEVs derived from uninfected vaginal cells (p = 0.0296 and p = 0.0015 for 347V+ and UR1 infection, respectively). This immunosuppressive effect was reversed when the 347V+ strain was cured from the virus. The sEVs from the 347V- infection phenotype induced similar activation as the sEVs from uninfected vaginal cells (p > 0.99). sEVs derived from vaginal cells exposed to poly(I:C) had a similar immunosuppressive effect (p < 0.0016) as the sEVs from the TVV-positive infection phenotype suggesting that the vaginal sEV manipulation may be at least in part attributable to direct effect of TVV dsRNA. IL-10 followed the same pattern of decreased expression in MALP-2-treated PBMCs as IL-8 (**Figure 3B**). The decrease in cytokine output occurred in the absence of cell toxicity (**Figure 3C**).

We then tested the effect of the vaginal infection sEVs on PBMCs in the presence of MALP-2, a TLR2/6 ligand derived from mycoplasma, another frequent TV endosymbiont (Fichorova et al., 2017). MTT data confirmed lack of toxicity under all experimental conditions (**Figure 3C**). As expected, and shown in **Figure 3A**, MALP-2 induced a 3-fold increase of IL-8 production by the PBMCs in the absence of exposure to sEVs from the vaginal infected cells. Exposure of the PBMCs to vaginal sEVs from the TVV+ infection phenotype significantly suppressed the IL-8 response to MALP-2 (MALP-2 alone versus combined with UR1+ and 347V+, p < 0.0164 and p < 0.0254, respectively). sEVs from vaginal cells activated with poly(I:C) showed similar immunosuppressive effect (p < 0.0022). Similarly to the effects of sEVs in the absence of MALP-2, the removal of the virus from the 347V+ strain reversed the immunosuppressive effect (347V- > 347V+, p < 0.0001) and 347V- led to a higher activation status compared to MALP-2 alone (p = 0.0014), similar to that induced by sEVs from uninfected vaginal cells.

The immune-suppressive effects of the sEVs of the TVV+ infection phenotype in both absence or presence of MALP-2 were reproduced with PBMCs from multiple donors (**Supplementary Figure S4**).

### Quantitative Proteomic Analysis of sEVs Derived From *T. vaginalis*

**Figure 4:**
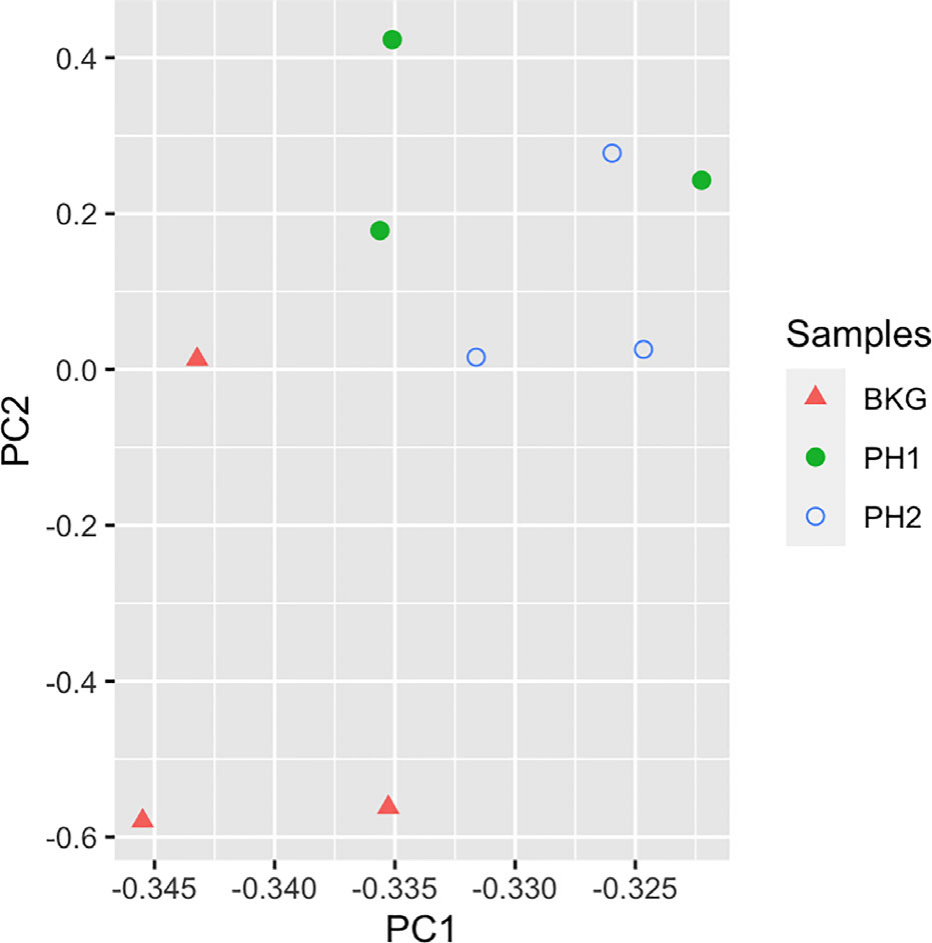
TV sEVs cluster separately from background medium control. Principal component analysis was used to compare sEVs on the log2-transformed abundance values of all proteins identified. Triangles represent background medium (BKG), filled circles represent virus-negative TV phenotype (PH1), unfilled circles represent virus-positive TV phenotype (PH2).

**Figure 5:**
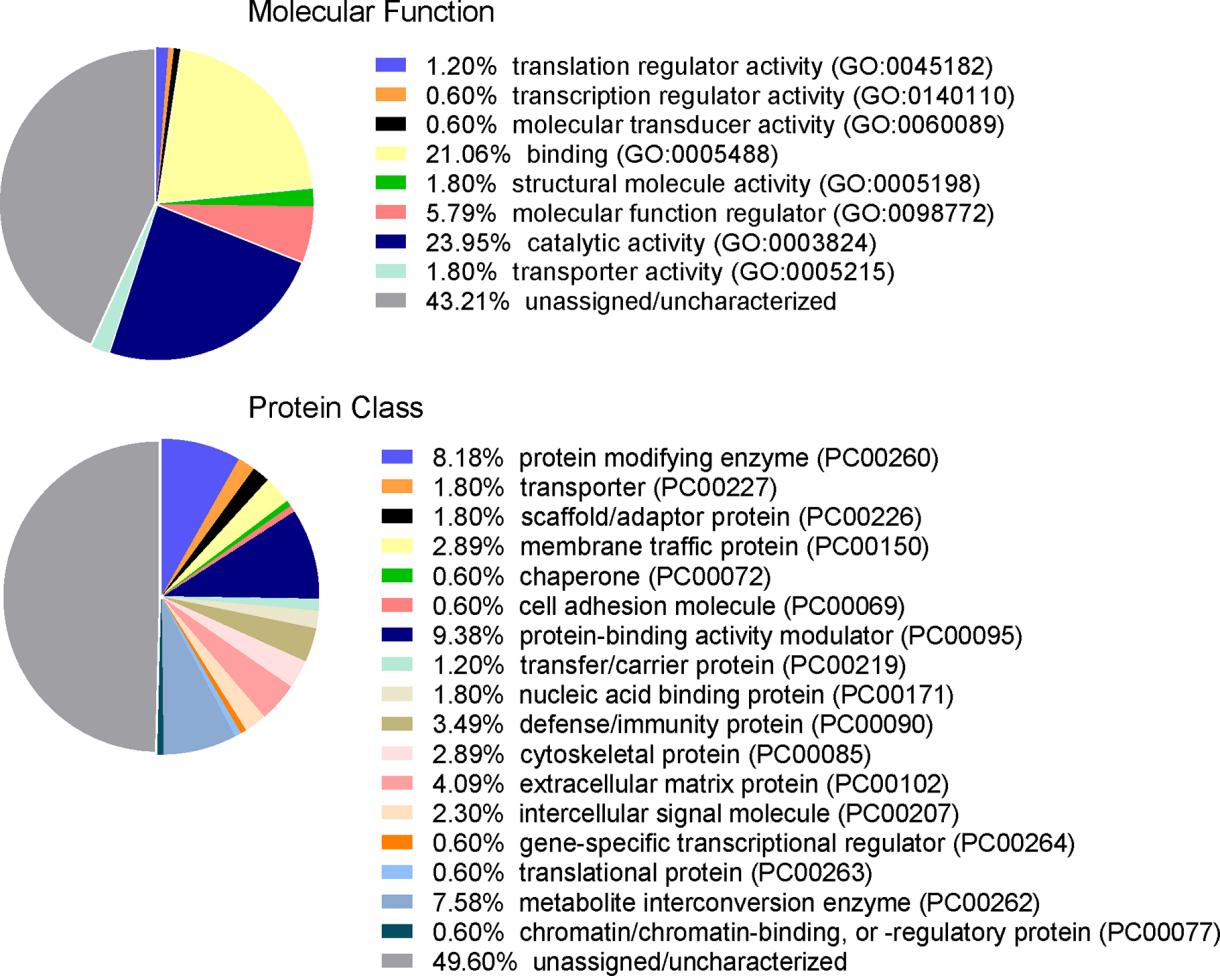
Functional categorization of TV sEV proteome. The identified TV sEV proteins were sorted into functional groups for molecular function with gene ontology (GO) terms and for protein class (PC) using PANTHER classification system (pantherdb.org).

**Figure 6:**
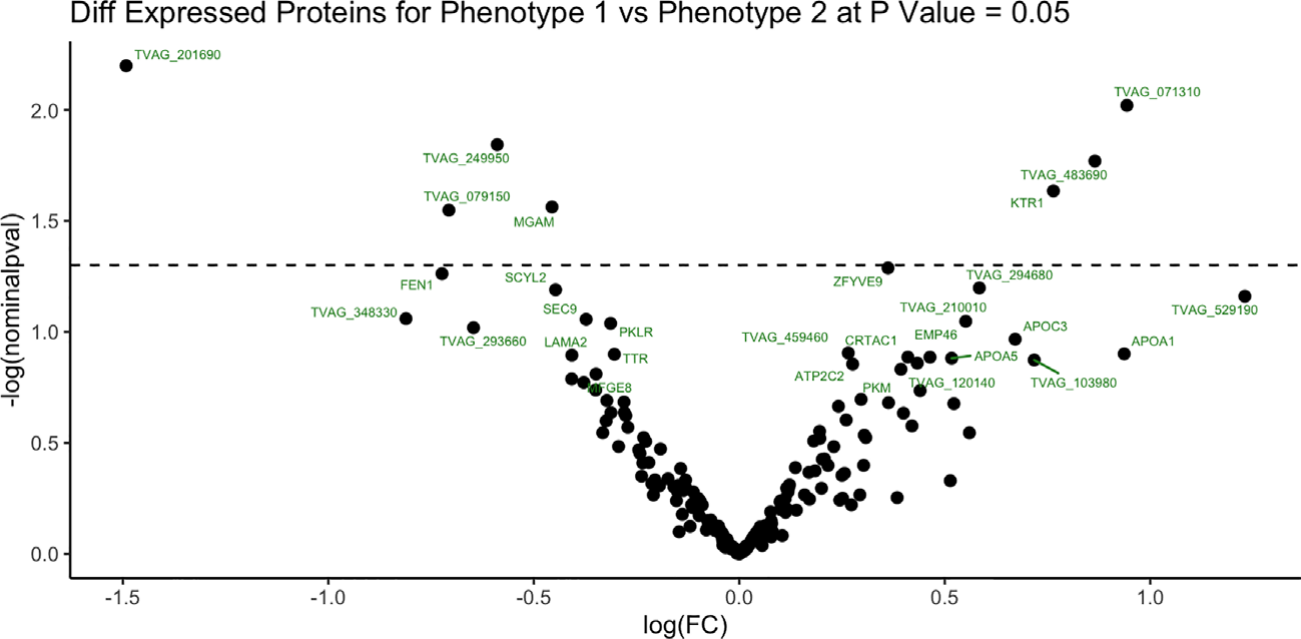
Differentially expressed sEV proteins between virus-negative TV vs. virus-positive TV. Differential expression analysis compared log2-transformed abundance values of all sEV proteins identified between virus-negative TV vs. virus-positive TV. Dashed line represents p value = 0.05. X-axis represents log2-transformed fold change (FC). Y-axis represents -log2 transformed P value.

**Figure 7:**
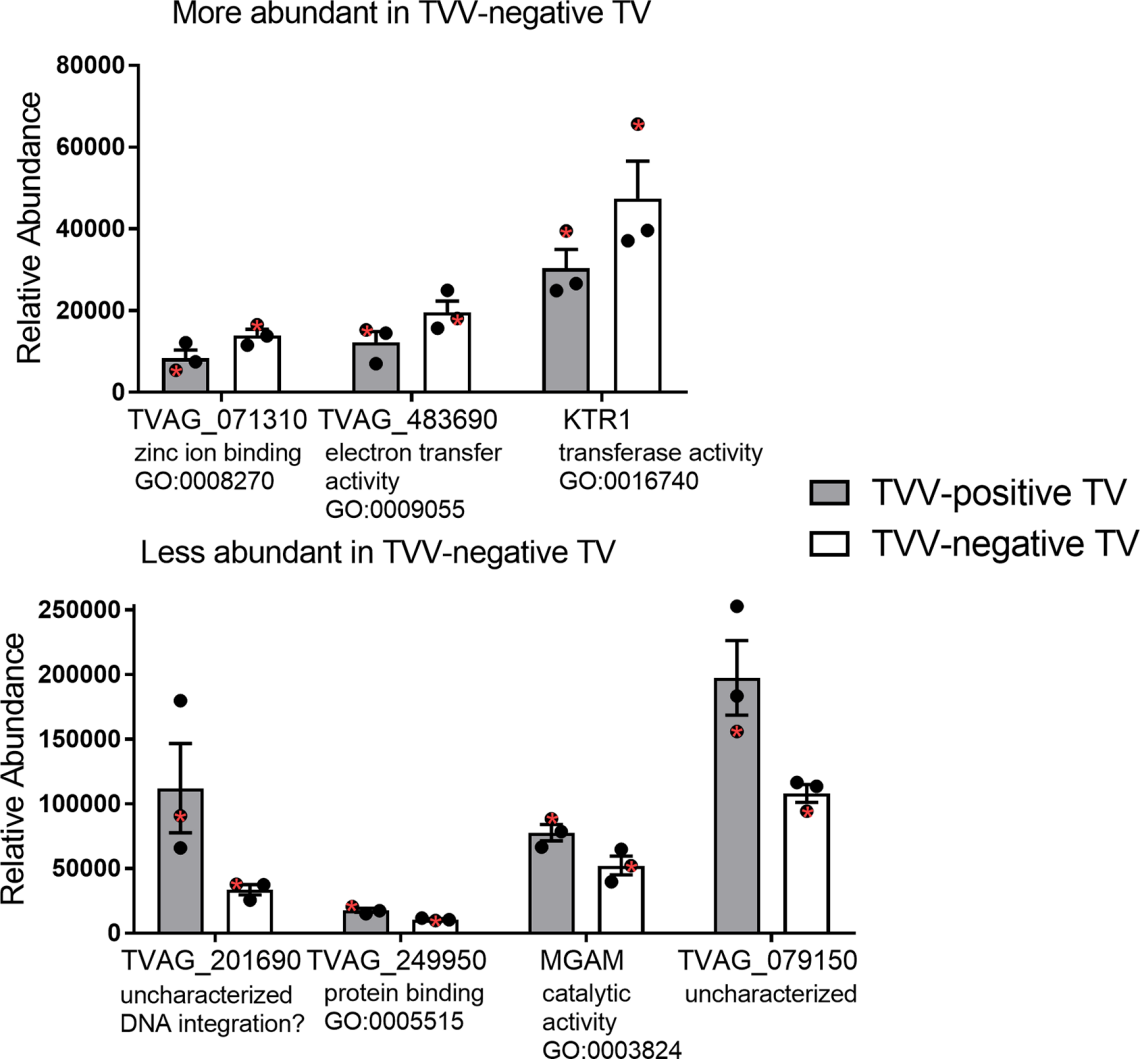
Relative abundance of the differentially expressed sEV proteins. Differential expression analysis compared log2-transformed abundance values of all sEV proteins identified between virus-negative TV vs. virus-positive TV. Graphs represent relative abundance values of the 7 significantly differentially expressed (p < 0.05) proteins between three virus-negative TV strains vs. three virus-positive TV strains and plotted as mean ± standard error of mean. The isogenic strains 347V+ and 347V- are noted with a red asterisk within each phenotype. For each protein, gene ontology (GO) terms for molecular function are annotated.

To identify proteins that may be involved in this differential immune response, we examined the expression of proteins packaged into sEVs released by virus-positive and virus-negative TV. Quantitative proteome analysis using TMT-labeling compared sEVs derived from three virus-positive TV strains (347V+, UR1 clone, and OC8) and three virus-negative TV strains (347V-, OC7, and B7RC2). The sEVs from the 6 TV strains clustered separately from the sEVs isolated from culture medium as a background control (**Figure 4**). A total of 241 proteins were identified with 192 consistently present in the sEV preparations from all 6 TV strains. Of those 11% (21 proteins) were identified as common contaminants. Of the remaining 171 proteins detected in the TV sEVs, 67% (114 proteins) were matched to the UniProt human database suggesting high homology with human proteins, 6% (11 proteins) were matched to the yeast database, and 27% (46 proteins) were identified as unique TV proteins with no homology to human or yeast. The 171 TV sEV proteins were characterized by molecular function and protein class according to the PANTHER classification system, which uses Gene Ontology (GO) (Mi et al., 2019). According to the predicted function assigned by GO; 23.95% are proteins involved in catalytic activity and 21.06% are involved in binding (**Figure 5**). In smaller proportion, assigned protein functions included molecular function regulators, transporters, structural molecules, transcription and translation regulators and molecular transducers. TV proteins categorized by protein class were assigned into 17 classes, with the larger proportions belonging to protein binding and modifying, metabolite enzyme, extracellular matrix, and defense/immunity. Majority of proteins identified were not assigned to a functional group and this could partly be due to a large number of TV proteins being uncharacterized. Differential expression analysis showed seven proteins were significantly differentially expressed (DE) (p < 0.05) between virus-negative vs. virus-positive TV (**Figure 6**). The relative abundance values of the DE proteins show that the protein expression data from the TV strains tightly clustered by TVV phenotype (**Figure 7**). According to UniProt database (Uniprot Consortium, 2018), the three proteins (TVAG_071310, TVAG_483690, and KTR1) that were more abundant in the virus-negative phenotype are involved in zinc ion binding (GO:0008270), electron transfer activity (GO:0009055), and transferase activity (GO:0016740), while the human protein MGAM that was less abundant in that phenotype is involved in catalytic activity (GO:0003824), metabolic process (GO:0008152), and neutrophil degranulation (GO:0043312). The other three proteins (TVAG_201690, TVAG_249950, and TVAG_079150) that were less abundant in the virus-negative sEV phenotype are considered uncharacterized hypothetical proteins. According to TrichDB genome database (Aurrecoechea et al., 2009), TVAG_249950 hypothetical protein has a predicted function of protein binding but no functional information is available for TVAG_079150 or TVAG_201690. Protein BLAST analysis (Uniprot Consortium, 2018) revealed TVAG_201690 shares sequence similarity to five different integrase core domain proteins from *Tritrichomonas foetus* and therefore may be involved in DNA integration, while TVAG_079150 shares sequence similarity to multiple uncharacterized proteins.

## Discussion

Our study is the first to show in human vaginal epithelial cells, the primary *T. vaginalis* host can release immunosuppressive sEVs in response to infection with TVV-positive TV and that these immunosuppressive effects are eliminated and replaced by immune stimulation upon cure of the parasite from the symbiont virus. It is also first to compare the sEV effects and their protein cargo in virus-positive versus virus-negative phenotypes derived from isogenic parasites.

Extracellular vesicles (EV) secreted by protozoan parasites have been shown to facilitate parasite-host as well as parasite-parasite communications and functions including parasite adherence, tissue tropism, drug resistance, and differentiation (Marti and Johnson, 2016; Wu et al., 2018). EVs originating from *Leishmania* spp., *Trypanosoma cruzi*, and erythrocytes infected with *Plasmodium* spp. have been implicated in immunomodulation, most of them showing upregulation of cytokines, while some, e.g., EV from *Leishmania* spp., causing downregulation of IL-8 and TNF-α in monocytes, but these phenomena were not investigated in the context of symbiont viruses (Szempruch et al., 2016). Our finding of suppressed IL-8 by sEVs from the TVV+TV infection may have clinical significance for the extracellular parasite adaptation since IL-8 is involved in the recruitment of neutrophils (Shaio et al., 1995)—the immune cells primarily responsible for clearance of TV parasites (Mercer et al., 2018; Bhakta et al., 2020).

Only a few studies to date have described sEVs released by TV, limited to parasites with either unknown or negative TVV status. A study by Twu et al. showed that sEVs released from TV upregulated IL-6 and IL-8 protein levels in human ectocervical epithelial cells in the absence of parasite, but IL-8 was lower when the cells were infected after sEV pre-incubation (Twu et al., 2013). Twu et al. listed several TV strains in the method section but did not specify, which one was used in each experiment and did not provide TVV status of the strains. Of note, the human ectocervical epithelial cell line used by Twu et al. is isogenic to the endocervical epithelial cell line in this study, both generated from the same female donor (Fichorova et al., 1997). A mouse infection model where sEVs from a TV strain GT-21 of unknown TVV status upregulated mRNA levels of IL-10, and slightly IL-6 and TNF-α in macrophages but sEV pre-treatment prior to TV infection showed mixed effects, e.g., increased IL-10, inhibited IL-13 and IL-17, and delayed inhibition of IL-6 (Olmos-Ortiz et al., 2017). Our study provided evidence that the symbiont virus is needed by the parasite to generate an immunosuppressive sEV phenotype. IL-10 is known for its immunosuppressive effects in other protozoan parasitic infections e.g. *Leishmania* (Kane and Mosser, 2001). However, our data suggest that the immunosuppressive effect of exosomes secreted by vaginal cells in response to TVV-positive TV and in response to the viral dsRNA mimic (poly-IC) occurs in the absence of IL-10 upregulation and, moreover, blunts IL-10 expression by PBMCs. In our study, the human PBMC’s responded to TLR2/6 stimulation by MALP-2 with IL-10 upregulation as expected. However, this IL-10 response was selectively suppressed upon exposure to sEVs from poly(I:C) (dsRNA) treated vaginal cells as well as vaginal sEVs from the TVV+TV but not the TVV-TV infection. Thus, mechanisms other than IL-10 are responsible for the immunosuppressive effects of circulating exosomes driven by viral dsRNA and virus positive TV phenotype. PBMCs isolated by the gradient method applied in our study typically contain 70–90% lymphocytes (T-cells, B-cells, and NK cells), 10–20% monocytes and 1–2% dendritic cells (Kleiveland, 2015). Thus, our findings primarily apply to the lymphocyte pool. Various cell types in the tissue context may respond differently as it is known that the IL-10 signaling depends on the cellular source (Iyer and Cheng, 2012). Since, we confirmed the immunosuppressive effect of the TVV-positive sEVs phenotype using multiple donors, it is unlikely that it is necessarily driven by T memory cells to TV proteins. The role of immune memory and cell types remains to be elucidated by future studies.

Various components of the sEV molecular cargo may mediate the observed immunomodulatory effects of the TV sEVs. Proteins, RNAs and episomal DNA have been found to mediate host-parasite interactions in protozoan infection models (Szempruch et al., 2016). One recent study showed that in a mouse infection model, *Leishmania* spp. can package their symbiont virus (LRV1) into EVs to facilitate viral transmission between the parasites which exacerbated the mucocutaneous lesions typical for the clinical presentation of leishmaniasis (Atayde et al., 2019). This study, however, did not measure cytokines and did not characterize other immunologic aspects of hijacking the *Leishmania* exosomal pathway by the virus. It is theoretically possible that whole TVV virions or genomic dsRNA may be carried by the TV sEVs. The TVV virions are at most 45 nm in diameter (Parent et al., 2013) and thus may fit into sEVs of ~100 nm size used in this study. We have, however, not observed them within our sEV preparations by electron microscopy (data not shown) suggesting that sEV-mediated transmission may be rare event in TV making it unlikely that the immunologic effects we observed are mediated by the uptake of virions wrapped in TV sEV membranes. The different ways protozoan-viral symbiosis exploits the sEV pathways in leishmaniasis and trichomoniasis may be explained by adaptation to different parasitic lifecycles. In contrast to TV, the *Leishmania* spp. are intracellular parasites that infect phagocytes relying on both myeloid and T cell responses for clearance of infection (Tripathi et al., 2007; Liu and Uzonna, 2012). In contrast to the role of virus in leishmaniasis, in trichomoniasis the viral symbiont does not appear to show a more severe symptomatology (Graves et al., 2019), which supports our hypothesis and findings that in TV the virus makes the host more permissive by blocking leukocyte responses that can clear the extracellular parasite.

It is also unlikely that the effects specific for the TVV-positive sEV phenotype were mediated by direct signaling of genomic viral dsRNA. The presence of such dsRNA in the sEVs if taken up by the endocervical epithelial cells by either membrane fusion or endocytosis would have caused a significant RANTES upregulation through the TLR3 signaling pathway as we have previously shown (Fichorova et al., 2012). However, this was not observed in our experiments when the cells were exposed to sEVs of the TVV-positive phenotype. In fact, the sEVs from TVV-negative strains induced RANTES upregulation while the sEVs from the TVV-positive phenotype were immunosuppressive. However, other pathways of dsRNA processing and effects may be involved since we were able to reproduce the immunosuppressive phenotype of the vaginal sEVs by stimulating with poly(I:C), a synthetic dsRNA mimicking genomic TVV in the absence of the parasite and the parasite-derived sEVs. Studies are under way to further investigate the role of RNAs including micro-RNAs in regulating the effects of sEVs generated in TV infection.

Prior research has presented evidence that TV sEVs contain proteins of significance for TV pathogenesis (Twu et al., 2013). Here we show for the first time that TV proteins packaged in the protozoan sEVs may depend on the endosymbiont status. Also, for the first time we report a quantitative comparative proteome analysis of sEVs originating from different TV strains. Our approach of using labeled proteomic analysis allowed us to quantitatively compare TV sEV proteomes in a mix of multiple virus-negative and virus-positive TV strains.

Our proteome analysis identified a total of 241 proteins and after comparison to the human and yeast databases and removal of common contaminants, 171 proteins were identified as consistently expressed by TV, with 114 shared with the human database and 46 uniquely matched to the TV database. Twu et al. identified a total of 215 proteins, with only 2 proteins that overlapped with those reported in this study. Multiple factors may account for these differences. The proteome analysis performed by Twu et al. was initial gel separation followed by multidimensional protein identification technology (MudPit), followed by a label-free quantitation of the proteome of a single strain. In contrast, we used a quantitative TMT labeled approach which allowed us to analyze a mix of three strains per phenotype (6 replicates). Firstly, differences in sample processing between these two methodologies may account for the differences in the number of proteins identified. The gel separation followed by MudPit mass spectrometry might lead to higher resolution and depth/coverage due to more fractionation and/or higher single sample input. In our study, we did not perform deep fractionation to achieve depth of the coverage but rather our goal was to show differences in sEV proteomes using labeling and triplicates in each group to have the statistical power to show proteins that differentiate between virus-negative and virus-positive TV phenotypes. Secondly, protein identification based on peptide sequences might have identified different proteins due to different databases screened. Both, our study and Twu et al. screened the same TV proteome database. However, our study also simultaneously screened the human and yeast databases, while Twu et al. did not report screening against those databases. Third, differences in TV growth conditions such as TV density, culture medium composition, and incubation time may account for differences in TV protein expression. In the study reported here, TV strains were grown in non-EV depleted Diamond’s medium to allow optimal growth conditions over a period of 24 h. In contrast, Twu et al. exposed TV strain to an EV-depleted TYM medium and harvested the sEV for protein analysis in only 4 h, allowing limited protozoan replication. Furthermore, in the present study, TV cultures were started at a 2.5-fold lower density to reduce stress derived from dying protozoa (4 × 10^5^ parasites/ml vs. 1 × 10^6^ parasites/ml by Twu et al), which could have also affected the sEV protein cargo.

Since protein identification in this study was done at the peptide-level, which may be indistinguishable by species, the 114 TV-expressed proteins identified as “human” are most likely TV proteins highly homologous to human and thus matched with higher confidence to the human proteome database which is much larger than the TV proteome database. The 46 proteins identified as TV were not homologous to human proteins and thus have unique peptides that belong to TV proteins only. Our findings suggest that 67% (114/171) of the sEV proteins expressed by TV are homologous to human proteins that are not common contaminants. These findings are in agreement with previous findings that TV exosome proteins contained 73% mammalian orthologs when compared to the ExoCarta database (Twu et al., 2013). Our analysis revealed that 54% (92/171) of the proteins identified in the TV sEVs were matched to proteins previously found in human exosomes described in the ExoCarta database (Keerthikumar et al., 2016). Similarly, another report showed a 56% proteome overlap between TV microvesicles and human EVs when compared to EVpedia (Nievas et al., 2018).

Our study showed enrichment for functions including catalytic, transcription, translation and transducer activities and protein classes including immune/defense proteins. Of the proteins identified as TV in our study, 30% (13/46 proteins) were previously identified in the TV secretome (Stafkova et al., 2018) and their functional relevance to host immunity is still to be elucidated. The single human protein (MGAM) more abundant in the virus-positive TV phenotype is involved in neutrophil degranulation. Neutrophils are important in resolving protozoan parasitic diseases (Mercer et al., 2018; Diaz-Godinez and Carrero, 2019) using killing mechanisms including degranulation whereby they release inflammatory mediators, proteases, and cytolytic factors (Lacy, 2006). Whether MGAM facilitates virus-positive TV sEVs in dampening the immune response should be investigated since it may have broader implications for neutrophil regulation. Less is known about the three TV proteins (TVAG_201690, TVAG_249950 and TVAG_079150) more abundant in the virus-positive phenotype and whether they may be involved in regulation of immune responses, therefore further investigation into the functions of these proteins is needed.

Another finding with important potential clinical implications is that sEVs derived from the vaginal infection with TVV-positive TV also suppressed signaling by a major mycoplasma protein (MALP-2). This phenomenon suggests that the parasite-viral symbiosis may dampen the immune surveillance against mycoplasma—another frequent symbiont of the TV parasite, which provides metabolic benefit to the parasite and facilitates the dysbiotic vaginal condition characteristic for trichomoniasis (Fichorova et al., 2017).

Collectively, our results suggest that endosymbiont virus-positive parasites may use the sEV pathways to evade host immunity. Future studies should continue dissecting the sEV cargo in the context of TV infection in symbiosis with *Trichomonasvirus* and mycoplasma to identify the molecular pathways of immune modulation and further implications for the human host.

## Data Availability Statement

The datasets generated for this study can be found in the MassIVE Repository (a member of the ProteomeXchange consortium), ID PXD021696.

## Ethics Statement

The studies involving human participants were reviewed and approved by Partners Human Research Affairs-Partners Human Research Committee, Brigham and Women’s Hospital. The patients/participants provided their written informed consent to participate in this study.

## Author Contributions

RF conceived the study. Data were generated by TC, YG, HY, and BB. Data analysis was performed by RF, YG, BB, and TC. The manuscript was drafted by YG and RNF. All authors contributed to the article and approved the submitted version.

## Funding

This study was supported by NIH/NIAID grants 1R56AI091889-01A1 and 1RC1AI086788-01 (RNF).

## Conflict of Interest

BB is a scientific adviser for Merck.

The remaining authors declare that the research was conducted in the absence of any commercial or financial relationships that could be construed as a potential conflict of interest.

## Supplementary Material

Supplementary Table 1 Proteomics data analysis. Data analysis sheets contain (i) the protein list of TV phenotype 1 (virus-negative) vs. phenotype 2 (virus-positive) with statistical analyses and includes UniProt accession number, gene symbol, log2 fold change (FC), FC, p-values and identification of common contaminants and (ii) the gene ontology (GO) analysis for protein class and molecular function using PANTHER classification system (pantherdb.org).
