# Supplementary material for: The Role of Small Extracellular Vesicles in Viral-Protozoan Symbiosis: Lessons From Trichomonasvirus in an Isogenic Host Parasite Model
Source: Front Cell Infect Microbiol. 2020 Nov 5;10:591172. doi: 10.3389/fcimb.2020.591172 (PMC7674494; doi:10.3389/fcimb.2020.591172)
Supplement: Supplementary file 1 [file DataSheet_1.docx]

Supplementary Material

## Supplementary Figures


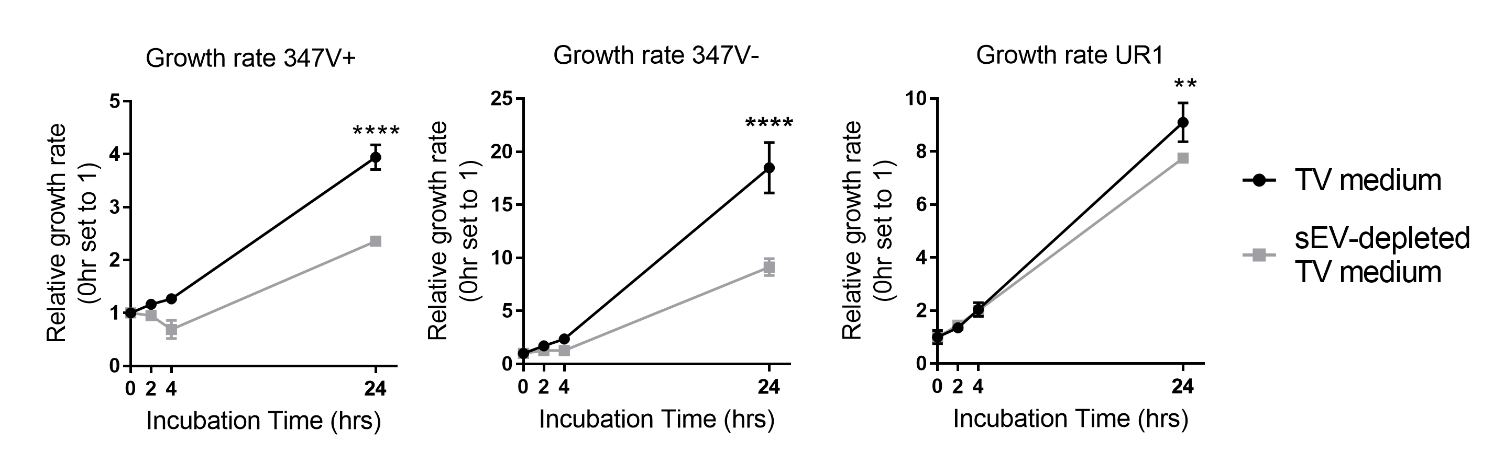


**Supplementary Figure S1. *T. vaginalis* growth is suboptimal in sEV-depleted TV medium.** TV parasites were grown in TV medium and sEV-depleted TV medium and growth was monitored for 0 - 24 hrs by live parasite counts represented as growth rate relative to 0 hr counts. Data represent three experiments performed in duplicate. Plotted values are means ± standard error of mean. Two-way ANOVA with Bonferroni’s post-test for multiple comparison was performed; **, p<0.01; and ****, p<0.0001 represent TV medium different from sEV-depleted TV medium.


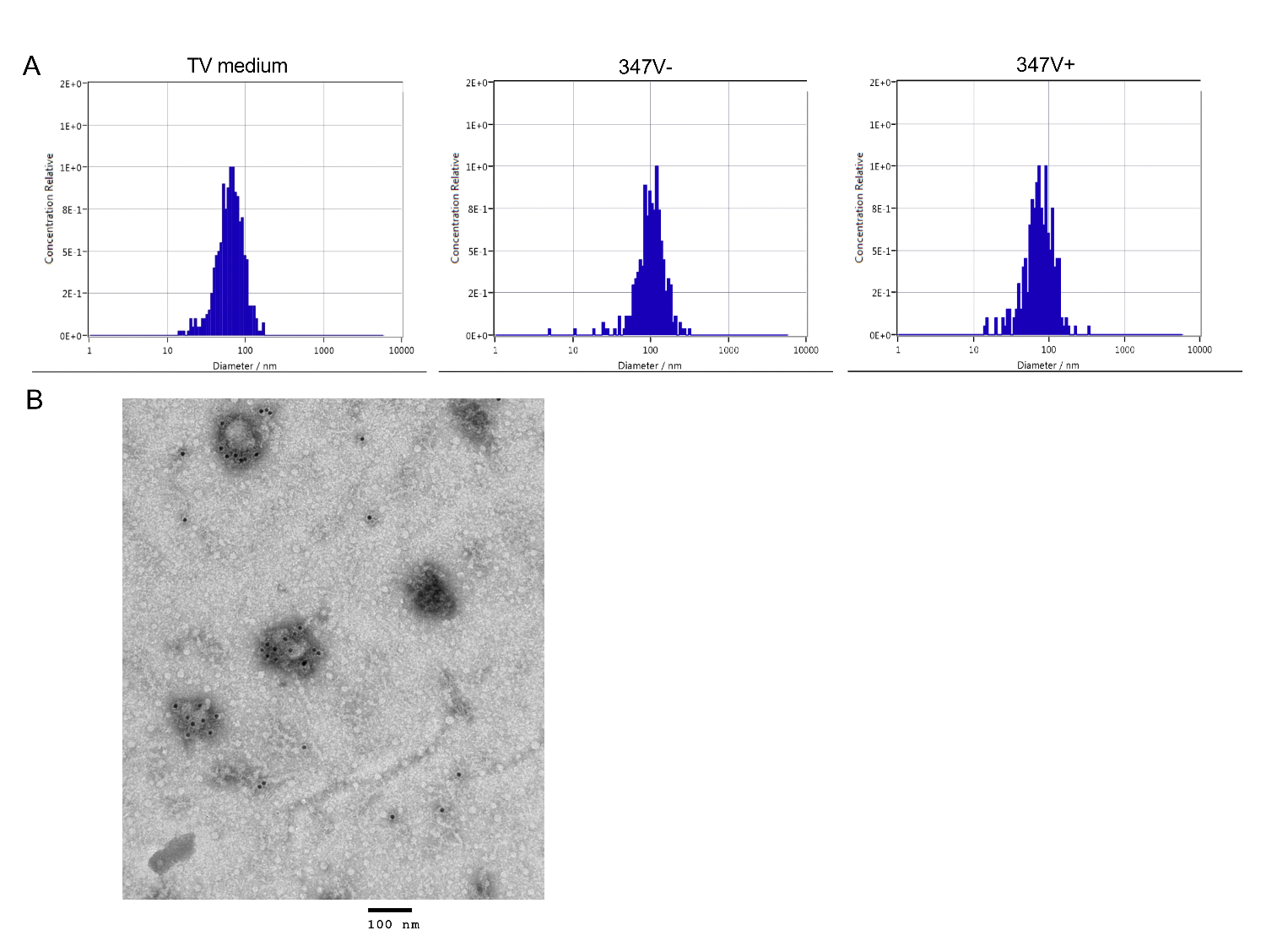


**Supplementary Figure S2. ZetaView nanoparticle tracking analysis and electron microcopy of sEVs derived from *T. vaginalis*. (A)** sEV samples were diluted in PBS (1000x – 50, 000x) and processed using video analysis of 2 data cycles in 11 scanning areas for a total of 22 measurements taken per sample. Representative images are shown of sEVs isolated from TV medium (1000x dilution), 347V- (1000x dilution) and 347V+ (50,000x dilution) with peak diameter of 67.4 nm, 106.9 nm and 76.6 nm and dilution corrected peak concentrations of 1.9E9, 1.1E9 and 1.65E11 particles/ml, respectively. **(B)** Transmission electron microscopy with immunogold labelling of CD63 in the sEV samples, a representative image is shown. Scale bar = 100 nm.


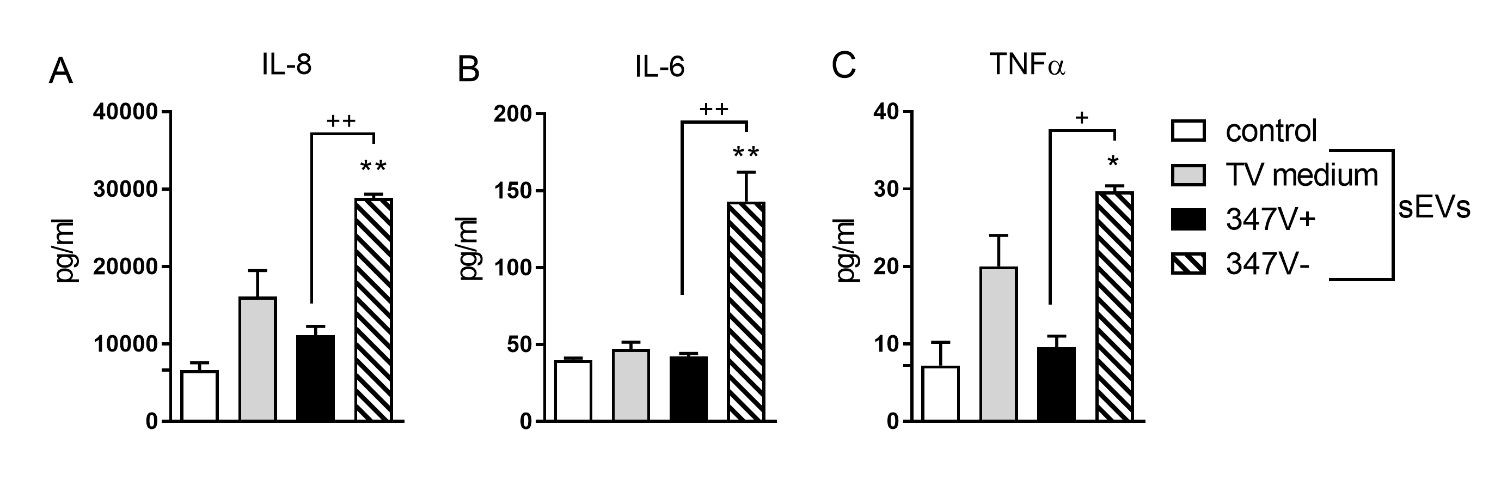


**Supplementary Figure S3. PBMC donor Y, sEVs from virus-negative TV but not virus-positive parasites induce multi-cytokine response in immune cells.** PBMCs were treated with control no sEV, sEVs derived from TV medium, 347V+ and 347V- TV strains for 24 hrs. **(A)** IL-8, **(B)** IL-6 and **(C)** TNF-α protein levels were measured by MSD assays. Graphs represent data from PBMC donor Y in duplicate and plotted as mean ± standard error of mean. Results are shown as protein levels. *, p<0.05 and **, p<0.01 represent sEVs different from control. +, p< 0.05 and ++, p<0.01 represent 347V- different from 347V+.


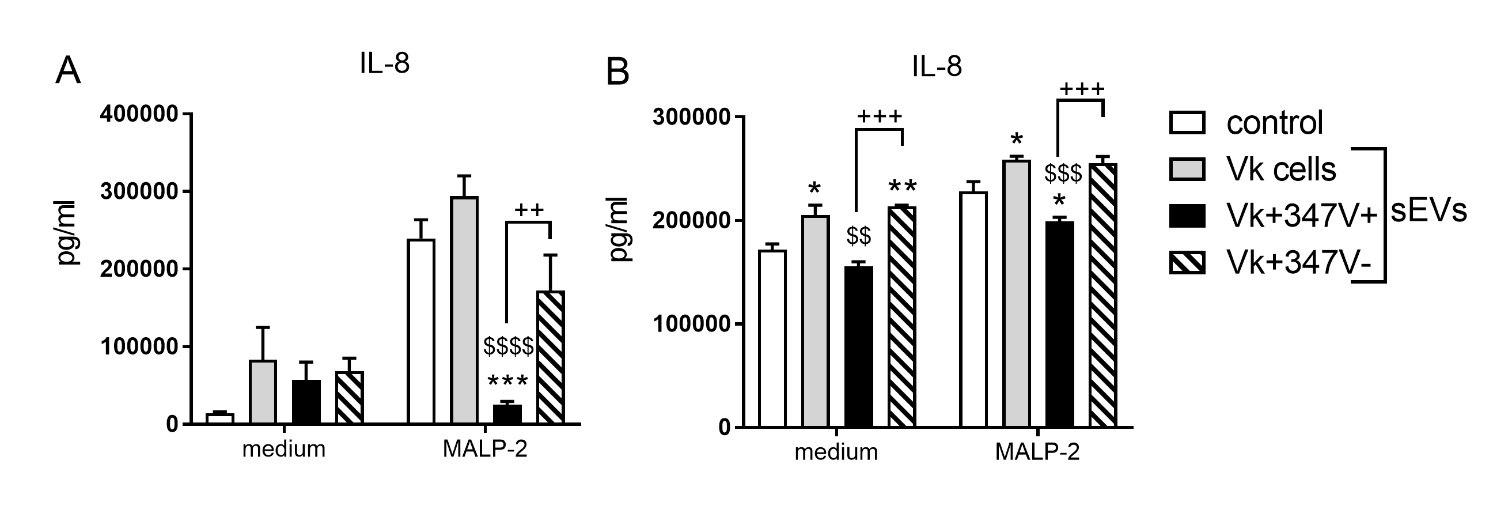


**Supplementary Figure S4. PBMC donor B and C, sEVs from TV-infected vaginal cells regulate cellular immunity dependent on viral status of the parasite.** PBMCs were treated with control no sEVs, with sEVs derived from vaginal cells (Vk cells), Vk+347V+ and Vk+347V- with or without MALP-2 for 24 hrs. IL-8 protein levels were measured by MSD assays. Graphs represent data from **(A)** PBMC donor B and **(B)** donor C in at least duplicate and plotted as mean ± standard error of mean. *, p<0.05; **, p<0.01 and ***, p<0.001 represent sEVs different from control. $$, p<0.01, $$$, p<0.001 and $$$$, p<0.0001 represent comparisons different from Vk cells sEVs. ++, p<0.01 and +++, p<0.001 represent comparisons different from Vk+347V- sEVs.
